# The impact of the major causes of death on life expectancy in China: a 60-year longitudinal study

**DOI:** 10.1186/1471-2458-14-1193

**Published:** 2014-11-20

**Authors:** Pengcheng Liu, Chengyue Li, Ying Wang, Wei Zeng, Hua Wang, Honghui Wu, Jun Lu, Mei Sun, Xiaohong Li, Fengshui Chang, Mo Hao

**Affiliations:** Research Institute of Health Development Strategies, Fudan University, Shanghai, 200032 China; Health and Family Planning Commission of Jiangsu Province, Nanjing, Jiangsu 210009 China; The Innovation Center for Social Risk Governance in Health, Shanghai, 200032 China

**Keywords:** China, Infectious and parasitic diseases, Chronic diseases, Accidental injuries, Maternal diseases, Impact, Life expectancy, Potential gains in life expectancy (PGLEs), Mortality data

## Abstract

**Background:**

In the 12th Five-Year Plan, the Chinese government set the goal of increasing life expectancy by one year. The purpose of this study is to examine the impact of major causes of death on the life expectancy of the Chinese people between 1950 and 2010 and predict changing trends to identify major issues requiring future attention.

**Methods:**

A continuous database organised by population and death data on diseases by age group between 1950 and 2010 were created from A Province in Eastern China. The diseases were classified into four categories by the International Classification of Diseases-10 (ICD-10): infectious and parasitic diseases, chronic diseases, accidental injuries, and maternal diseases. Potential gains in life expectancy (PGLEs) were applied to reflect the impact on life expectancy caused by deaths from various diseases, by using the cause-eliminated life table.

**Results:**

The PGLEs of infectious and parasitic diseases decreased from 15.59 years in 1950, to 0.07 year in 2010, and have remained low since 2000. However, the PGLEs of chronic diseases increased from 8.70 years in 1950, to 13.36 years in 2010, and indicated an increasing future trend. The two opposite trends exhibited a ‘scissors-like difference’. The proportion of accidental injuries and maternal diseases in the death spectrum was low. The PGLEs of accidental injuries decreased from 2.95 years in 1950, to 0.86 year in 2010, maintaining a low level, while the PGLEs of maternal diseases dropped from 0.56 to 0.002 year during the same period, approaching zero.

**Conclusions:**

The findings of this study provide useful information, which could contribute to a more effective allocation of public health programmes. In recent years, chronic diseases and accidental injuries have emerged as major factors influencing life expectancy. Primary and secondary prevention actions, such as public education, modification of behaviours, and introduction of safety measures should be emphasised in efforts to promote life expectancy. The morbidity and mortality rates of infectious, parasitic, and maternal diseases should be maintained at low levels.

## Background

Life expectancy provides an estimate of the average expected life span under certain conditions, based on current mortality. It is one of three comprehensive indicators reflecting an individual’s health, social and economic status, and quality of life [1]. It is also the most representative and comprehensive index to judge the social economy and healthcare development of a country or region [2]. Therefore, it has been incorporated gradually into the evaluation system of governmental performance. Since the founding of the People’s Republic of China (PRC) in 1949, China has markedly improved the health of its people. The life expectancy of Chinese residents has more than doubled, from 35 years of age in 1950, to 74.8 in 2010. The maternal mortality rate has decreased from 1,500/100,000 to 30/100,000, and the infant mortality rate has dropped from 200‰ to 13.1‰ [3].

However, changes in living conditions and lifestyles due to the aging of the population and urbanisation and industrialisation have changed China’s disease and death spectrum. The United Nations predicted that China’s elderly population (over 65 years) would reach 133 million by 2015, 169 million in 2020, and 197 million in 2025 [4]. The Fourth National Health Services Survey of China [5] reported that the morbidity rate of chronic diseases was 19.99% in 2008, indicating an increase of 10 million new cases on average, every year during the past decade, while the morbidity rate of infectious diseases dropped to 0.27%. According to the Third Death Cause Retrospective Review in 2005, infectious diseases and maternal and infant diseases that accounted for the proportion of total deaths dropped from 27.8% to 5.2%, while chronic diseases rose from less than 50% to 82.7% during 1973–2005. Today, China’s disease and death spectrum places chronic diseases as the major cause of death, with some of the major infectious diseases continuing to threaten people’s health [6, 7].

The Chinese government has attached great importance to the improvement of the population’s health. In the 12th Five-Year Plan of China [8], the government set the goal of increasing life expectancy by one year. To achieve this goal, reasonable health policies based on preliminary measures must be developed to understand the major issues.

The purpose of this study is to examine the impact of the major causes of death on the life expectancy of the Chinese people and the changing trends in their health status between 1950 and 2010, which should allow us to provide a clearer perspective of their impacts on life expectancy. We also used historical and current data to predict health trends and changes to identify major issues requiring future attention.

Previous studies [9–13] have used life tables with potential gains in life expectancy (PGLEs) as a research method to determine the impact of fatal diseases that cause death on the life expectancy of a population. However, most of these studies have focused on annual data or data from short time-spans, and do not reflect trends of the impact over longer periods or predict future trends. This study uses the PGLE method that eliminates certain causes of death to determine life expectancies in their absence, and focuses on a 60-year time span. This approach should allow us to reflect clearer trends of the impact of the major causes on life expectancies.

## Methods

### Study setting

We planned to collect population data on deaths caused by various diseases for each year from 1950 to 2010, by age group. However, it was difficult to obtain nationwide data that was continuous and accurate by age group because of the limited information on disease control and prevention, and lags in reporting deaths and disease to the registration system. In addition, it was difficult to coordinate the process of data collection because the management of the population’s disease mortality data belongs to different departments of the government.

We selected A Province, which is located in the Eastern Region of China, as the research setting. It is a relatively well-developed area compared to the Middle and Western regions. In 2010, A Province’s per capita GDP was 52,664 RMB, and it was ranked fourth among the eight provinces of Eastern China, in per capita GDP [14]. The health developments of A Province exhibited rapid progress in the past 60 years since China was founded [15], and data such as death rates due to diseases have been well preserved. Additionally, our study received support from the Health Department of A Province.

### Measures

#### Potential gains in life expectancy (PGLEs)

Based on the theory of competing risks and the recommendations generated from previous research studies, we selected PGLE, which eliminates certain causes of death as the indicator of their impact on life expectancy. This method permits the analysis of the impacts of several causes of death on the average lifetime of the individuals in a population, taking into account the competing risks acting among them [16]. It has been cited as an ideal method for this type of research [17].

As a quantitative measure, PGLEs can reflect the years of life lost resulting from deaths caused by a certain disease in a specific age group of the population [11]. It is defined as the number of additional years that a person of a certain age would expect to live on average, if the specific cause of death were eliminated [13]. The basic assumption is that if a particular disease that causes deaths is eliminated; those who might have died from the diseases live longer, thereby prolonging the life expectancy of the population. We called the prolonged life expectancy caused by the elimination of deaths from certain diseases the cause-eliminated life expectancy, which can be calculated by building a cause-eliminated life table. The PGLE of a disease in a certain year is calculated as follows: assuming that the cause-eliminated life expectancy is *e*_*x*_ and the life expectancy in the presence of all causes of death in the same year is *E*_*x*_, then the PGLE in this year, Δ*E*_*x*_ is


If we use the calculation of the PGLE of heart disease in A Province in 2010 as an example, then the PGLE of heart disease equals the cause-eliminated life expectancy of heart disease *e*_2010_ minus the life expectancy of 2010 *E*_2010_, yielding a result of 1.68 years.

If we eliminate a larger threat to the population’s health, the life expectancy will be extended further and the PGLE will be larger. Therefore, the PGLE is a reasonable way to explain the impact of a certain disease on life expectancy because it can reflect the loss of life expectancy caused by a certain disease and provide a numerical indicator of survival if the disease is eliminated. In addition, this indicator is not affected by the age structure of the population, facilitating comparisons between diseases [18].

#### Classification of diseases

Based on the Tenth Revision of the International Classification of Diseases (ICD–10), this study classified the main causes of death into four categories: infectious and parasitic diseases, chronic diseases, accidental injuries (injury and poisoning), and maternal diseases. Although the proportion of deaths due to maternal diseases has been low, the maternal mortality rate was very high worldwide in the early years of the PRC, and declined noticeably between 1950 and 2010. Moreover, the maternal mortality rate is one of three indicators commonly used to evaluate a population’s health. Therefore, we assessed the impact of maternal diseases on life expectancy in this study.

Maternal diseases include the complications of pregnancy, childbirth, and the puerperium. Chronic diseases include tumours, haematopoietic organs and immune diseases, mental disorders, nervous system diseases, circulatory-system diseases, respiratory diseases, digestive system diseases, musculoskeletal and connective tissue diseases, and genitourinary system diseases. This study also analysed diseases with high morbidity and mortality rates in China, such as tumours, circulatory-system diseases (including cerebrovascular disease, heart disease, and high blood pressure), and diabetes, accounting for nearly 80% of all deaths among the entire population in 2010.

### Data sources

We planned to collect population and death data (1950–2010) on the diseases by age group of A Province, according to the basic requirements of the cause-eliminated life table. Age was categorised into the following 19 groups: <1, 1–4, 5–9, 10–14, 15–19, 20–24, 25–29, 30–34, 35–39, 40–44, 45–49, 50–54, 55–59, 60–64, 65–69, 70–74, 75–79, 80–84, and >85.

The population data were derived from the ‘Statistical Yearbook of A Province’ [19], which contains census data. The mortality data on infectious and parasitic diseases before 2004 were taken from the ‘Health Statistical Data Collection of A Province’ and the ‘Acute Infectious Disease Epidemic Information Collection of A Province’, and after 2004, were taken from the Direct Network System of Infectious Diseases in China. The mortality data on chronic diseases, accidental injuries, and maternal diseases were obtained from the ‘Health Statistical Data Collection of A Province’.

Since several years of data from 1950 to 2010 were missing, these data were simulated through a time series model based on historical data. Finally, we created a continuous database that spanned 60 years.

### Data analysis

We created a database of different disease types in Excel 2010. Data analysis and time series modelling were conducted using Excel 2010 and Eview software. The PGLEs were calculated from the life table and the cause-eliminated life table.

## Results

### Trends of life expectancy and disease mortality

In the past 60 years, the life expectancy and disease mortality of A Province changed substantially. The mortality rates of infectious and parasitic diseases decreased from 848.3/100,000 to 5.2/100,000; accidental injuries from 177.1/100,000 to 39.8/100,000; and maternal diseases from 280.3/100,000 to 6.24/100,000. However, the mortality rates of chronic diseases increased from 391.2/100,000 to 691.3/100,000. The life expectancy of A Province increased markedly from 51.9 to 78.5. It should be noted that tumours and circulatory-system diseases accounted for most of the deaths due to chronic diseases. For example, in 2010, the mortality rates, respectively, of tumours and circulatory-system diseases were 193.13/100,000 and 268.86/100,000 (cerebrovascular disease 151.75/100,000 and heart disease 110.14/100,000).

### Historical trends of PGLEs from 1950–2010

**Figure 1:**
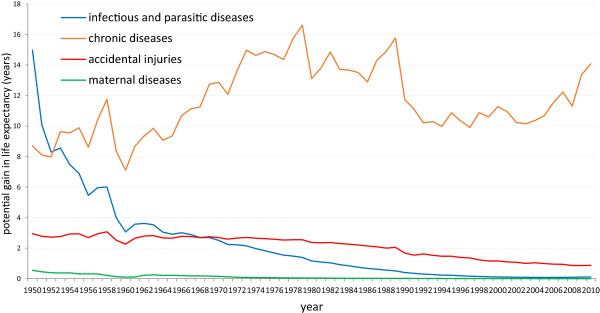
**Historical trends of PGLEs, diseases, and accidental injuries (1950–2010).**

In 1950, the PGLEs of infectious and parasitic diseases, chronic diseases, accidental injuries, and maternal diseases were 15.59, 8.70, 2.95, and 0.56 years, respectively, in A Province (Figure 1). In the years shortly after the founding of the PRC, the PGLEs of infectious and parasitic diseases were the largest, followed by chronic diseases, accidental injuries, and maternal diseases. At the time, the nutrition, living environment, and health conditions of the residents were very poor. Infectious and parasitic diseases were widespread, which led to high death rates in the population. At the same time, the mortality rates of chronic diseases and accidental injuries were very low.

However, with improvements in disease control and prevention, living environment, and nutritional status, the morbidity and mortality rates of infectious and parasitic diseases declined rapidly. The PGLEs also decreased from 15.59 years in 1950, to 0.07 year in 2010, and have remained low in recent years since 2000. In contrast, the PGLEs of chronic diseases increased from 8.70 years in 1950, to 14.07 years in 2010, and have exhibited an increasing trend because lifestyle changes have led to rising morbidity and mortality rates of chronic diseases. The two opposite trends in morbidity and mortality are evidenced by the ‘scissors-like difference’ between the PGLEs.

Compared to the sharp changes in the trends of infectious and parasitic diseases and chronic diseases, the proportion of accidental injuries and maternal diseases in the death spectrum is low. In recent years, mortality rates dropped annually because of positive prevention and control measures. The PGLEs of accidental injuries decreased from 2.95 years in 1950, to 0.89 year in 2010, maintaining a low level, while the PGLEs of maternal diseases dropped from 0.56 to 0.0018 in the same period, approaching zero.

This trend suggests the need to develop measures to prevent and control chronic diseases and accidental injuries as a way to improve people’s life expectancy, while maintaining low death rates due to infectious, parasitic, and maternal diseases.

**Figure 2:**
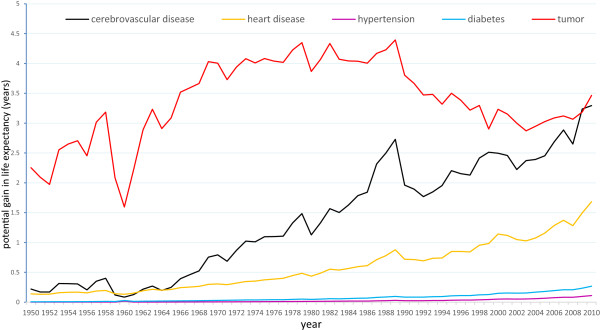
**Historical trends of PGLEs, the major types of chronic diseases (1950–2010).**

We further examined the PGLEs of several types of chronic diseases in order to understand the impact of the major chronic diseases on life expectancy (Figure 2). The trends in the PGLEs of the major chronic diseases were similar to the entire group of chronic diseases. The diseases with high morbidity rates, such as cerebrovascular disease, heart disease, hypertension, and diabetes, showed an increasing trend year by year. The rising rate of cerebrovascular disease is the largest, from 0.219 to 3.294 during 1950–2010. The PGLEs of hypertension changed the least, from 0.0004 to 0.097; heart disease and diabetes increased to a lesser extent.

Unlike the trends of the entire group of chronic diseases, the control of tumours improved in the past 30 years. The PGLEs of the tumours gradually increased at the beginning, but flattened out and even decreased in recent years. This trend also reflects the death trends in the major types of chronic diseases in the population.

### Predicting trends of PGLEs for 2011–2020

Based on the trends in total mortality and the mortality of each age group during 1950–2010, we used time series modelling to fit the trend curve of disease mortality (goodness of fit >0.8). We predicted the disease mortality trend in each age group during 2011–2020 in accordance with the fitting curve. Then, we calculated the PGLEs of different types of diseases using the cause-eliminated life table from 2011 to 2020, which were regarded as ‘the natural trend of PGLEs’. However, if public health efforts changed in controlling and preventing disease, the trends in the PGLEs would also be expected to change. This study predicted future PGLEs when prevention and control efforts changed, using the mortality of different types of diseases as the index of control. We assumed a certain percentage decline in the death rates because of stronger prevention and control measures or a certain percentage rise in the death rates resulting from weaker prevention and control efforts. We used stronger prevention and control measures to project future PGLEs in the following example. Based on the fitting curve of the disease mortality trend during 2011–2020, we assumed that the mortality rate of a disease in 2020 was A, under the natural trend, and that stronger prevention and control measures led to a decline in the mortality rate by X%. Then we calculated PGLEs using the cause-eliminated life table assuming a mortality rate of A*(1-X%), which would be the result when prevention and control efforts were increased. The decrease and increase percentages in the death rates were determined by consultation with several practice experts from public health centres. Based on their work experience, the consultants suggested an interval for the changes in the percentages, with a 10% increase as the upper limit and a 10% decrease as the lower limit. In this study, we used a 10% change in the death rate as an example to show the trend changes in the PGLEs.

If the intensity of public health efforts to prevent and control the rate of chronic diseases remains unchanged from 2011 to 2020, PGLEs will continue to increase to 18.96 years in 2020 (Table 1). Likewise, if the intensity strengthens or weakens by 10%, PGLEs will be 17.06 or 20.85 in 2020. There will be large differences in PGLEs when natural trends are compared with stronger and weaker efforts. If the current intensity of efforts to prevent and control accidental injuries does not change during 2011–2020, then the PGLEs will continue to decrease to 0.657 year by 2020; and if the efforts increase or decrease by 10%, the PGLEs will be 0.617 or 0.707 year. However, the PGLEs of infectious, parasitic, and maternal diseases will gradually shrink between 2011 and 2020, reaching a very low level. Even if prevention and control measures are increased or decreased by 10%, the PGLEs are expected to show little, if any, change. This forecast suggests that chronic diseases will be the major diseases affecting life expectancy in the future.

**Table 1:** **Predicting outcomes of the PGLEs for accidental injuries and different types of diseases in 2020**

Disease types	Natural trend	Stronger prevention measures (a 10% decline in the mortality)	Weaker prevention measures (a 10% rise in the mortality)
Infectious and parasitic diseases	0.0386	0.0347	0.0424
Chronic diseases	18.96	17.06	20.85
Accidental injuries	0.657	0.617	0.707
Maternal diseases	0.000048	0.000043	0.000053

The current trends suggest that the PGLEs for cerebrovascular disease will gradually increase during 2011–2020, reaching 7.323 years in 2020 (Table 2). If the prevention and control efforts related to this disease increase or decrease by 10%, the PGLEs will be 6.018 or 8.929 years. The PGLEs for heart disease show the same trend, reaching 3.150 under the current trend in 2020 and approaching 2.739 or 3.597 when disease prevention and control are increased or decreased. The change is smaller for hypertension with PGLEs expected to reach only 0.273, 0.245, and 0.302, with current, strengthened, or weakened efforts of the public health system.

**Table 2:** **Predicting outcomes of the PGLEs for the major types of chronic diseases in 2020**

Disease types	Natural trend	Stronger prevention measures (a 10% decline in the mortality)	Weaker prevention measures (a 10% rise in the mortality)
Cerebrovascular disease	7.323	6.018	8.929
Heart Disease	3.150	2.739	3.597
Hypertension	0.273	0.245	0.302
Diabetes	0.670	0.603	0.738
Tumours	4.175	3.737	4.639

The PGLEs for diabetes and tumours will increase to 0.670 and 4.175, respectively, in 2020. Thus, cerebrovascular diseases and tumours will become the major diseases affecting people’s life expectancies in the future.

## Discussion

Between the years 1950 and 2010, the PGLEs of infectious, parasitic, and maternal diseases in China continued to decline while those of chronic diseases showed an upward trend. The PGLEs of accidental injuries remained at a high level while exhibiting a downward trend. It is estimated that the major diseases will continue their previous trends under natural tendencies between 2011 and 2020. If prevention and control efforts directed at different diseases are increased to the same extent, their effects on chronic diseases and accidental injuries should improve while their effects on controlling infectious, parasitic, and maternal diseases should not change significantly.

The PGLEs of infectious and parasitic diseases showed a downward trend because the Chinese government strengthened disease prevention and control measures, such as implementing infectious disease surveillance and planned immunisations. Measures taken to eliminate the development and spread of many infectious diseases gradually resulted in a steady decline in mortality [20, 21]. However, some diseases previously under control, such as measles and polio, show signs of returning in recent years. Therefore, strategies to control infectious and parasitic diseases should maintain high standards in programmes unremittingly. Preventing the return of infectious diseases that are under control, and the spread of emerging infectious diseases are also a high priority.

The reasons for the decrease in maternal mortality are similar to the reasons for the decrease in infectious and parasitic diseases. In the early days of the PRC, the maternal mortality rate was high because of lack of medicine and harsh childbirth conditions. As maternal health measures were gradually implemented [22, 23], hospital delivery was standardised and popularised, comprehensive prevention and care measures for high-risk pregnant women were drastically improved, and the maternal care of the migrant population was strengthened, with the maternal mortality rate showing a steady downward trend. However, the maternal health of the underdeveloped Western Regions continues to be poor, which also requires more attention in the future [24].

In the early years of the PRC, the mortality rate of chronic diseases was relatively low. However, due to the gradual control of infectious, parasitic, and maternal diseases, acceleration of China’s urbanisation and industrialisation, rapid growth of environmental pollution and occupational hazards, and changes in people’s lifestyles and working conditions, an increase in the prevalence of chronic diseases occurred, and deaths caused by chronic diseases gradually increased, affecting life expectancy.

In terms of the major types of chronic diseases, the impact of the circulatory-system diseases (including cerebrovascular disease, heart disease, and high blood pressure) and diabetes on life expectancy is increasing year by year. The impact of cancer on life expectancy also shows a rising trend, and this situation will continue for the next few years. Therefore, the prevention of circulatory system diseases and cancer are important considerations in future plans to improve people’s health. The modification of behaviours, which have been found to be associated with these specific pathologies, such as dietary habits, physical activity and smoking habits, should be improved.

Although deaths resulting directly from hypertension make a small contribution to the decrease in life expectancy, hypertension is a risk factor for many other diseases. Many patients with hypertension usually suffer from heart disease, cerebrovascular disease, and diabetes. According to one study, the risk attributed to hypertension on mortality resulting from a stroke in the Chinese population is 50% [25]. Therefore, we must attach great importance to the prevention and control of hypertension.

An analysis of tumours by age group found that the mortality of the younger groups is rising, suggesting that deaths caused by tumours tend to be in the younger population. Hence, the targeted population for cancer prevention and control efforts needs to be expanded. Early diagnosis is important in cancer prevention, and secondary preventive actions, such as screening programs can be extended to include younger people in order to achieve a reduction in mortality.

The PGLEs related to accidental injuries is decreasing because the overall injury mortality rate is declining, which has been corroborated by similar studies [26]. Although the overall mortality due to accidental injuries decreased, deaths due to motor vehicle accidents have increased sharply [27]. This finding suggests the need to focus on trends in specific types of injuries, in addition to bringing the overall injury mortality rates under control. Many traffic accidents are preventable by information and education; therefore, taking action to control alcohol abuse and the introduction of safety measures (seat belts, helmets, and child seats) are the most effective solutions [16].

This study had several limitations. First, we were unable to access the nationwide database due to its limited availability, and were only able to examine A Province as a sample. Because of the differences in socioeconomic development among the various provinces of China, the results and conclusions of this study might reflect the trends in the well-developed areas of Eastern China, while extrapolations to the entire country must be made carefully.

When making predictions of PGLEs under different levels of disease control and prevention, we assigned disease mortality as an indicator. Whether there are better indicators to guide the actual work is worth exploring.

## Conclusions

It must be pointed out that the hypothesis of the complete elimination of certain diseases that cause death is not realistic. However, we can evaluate the impacts of the diseases on life expectancies and assign the correct weights to the competing risks among the diseases. In the context of a nation’s public health, this approach is helpful for making health polices, developing goals, and allocating resources.

In the early stages of the PRC, infectious and parasitic diseases had a huge impact on life expectancy. With the implementation of health improvements, infectious and parasitic diseases were gradually controlled, and the morbidity and mortality rates of maternal diseases were reduced. In recent years, chronic diseases and accidental injuries have emerged as the major factors influencing life expectancy. With current trends, life expectancy would increase by 0.86 year in the next five years according to our projections, which means the goal of increasing life expectancy by one year could not be reached. Therefore, the prevention and control of chronic diseases and accidental injuries should be emphasised in efforts to promote life expectancy to achieve the goal. We should pay more attention to circulatory-system diseases, tumours, and diabetes. For infectious, parasitic, and maternal diseases with low morbidity and mortality rates, the priority is to maintain these low levels.

## Authors’ information

Pengcheng Liu and Chengyue Li are co-first authors.

## Abbreviations

ICD-10: International Classification of Diseases-10
PRC: the People’s Republic of China
PGLE: Potential gain in life expectancy.
